# Effect of pretreatment by supercritical fluids on antioxidant activity of protein hydrolyzate from quinoa (*Chenopodium quinoa* Willd.)

**DOI:** 10.1002/fsn3.2027

**Published:** 2020-12-03

**Authors:** Luis Olivera‐Montenegro, Ivan Best, Alonso Gil‐Saldarriaga

**Affiliations:** ^1^ Grupo de Ciencia, Tecnología e Innovación en Alimentos, Facultad de Ingeniería, Carrera de Ingeniería Agroindustrial Universidad San Ignacio de Loyola Lima Peru

**Keywords:** antioxidant activity, enzymatic hydrolysis, protein hydrolyzate, quinoa, supercritical fluid

## Abstract

The effect of two pretreatments on the antioxidant activity was evaluated in quinoa protein hydrolysate, using supercritical CO_2_ extraction and ethanol as cosolvent, this type of pretreatment was compared to a conventional petroleum ether extraction method without recovery of bioactive compounds. The extractions were carried out at a temperature of 55°C and a pressure of 23 MPa using ethanol (7–8 g quinoa/100 ml); the CO_2_ mass flow was 35 g/min, the extraction time was 240 min and the particle size was 500 µm, enzyme COROLASE^®^ 7089 was applied for enzymatic hydrolysis, finally ABTS test assessed antioxidant activity. A significant effect was found on the degree of hydrolysis (23.93%) and antioxidant activity (1,181.64 μmol TE/g protein) compared to a conventional method (24.33%) and (1,448.84 μmol TE/g protein). In conclusion, our results suggest that the use of supercritical CO_2_ and the addition of ethanol as cosolvent are the interesting green technology, to recovery oil and separate phenolic compounds prior to enzymatic hydrolysis to avoid interference with biological activities from quinoa protein hydrolysates, and shows highest antioxidant activity to be incorporate in food products.

## Graphical abstract

Separate phenolic compounds to avoid interference on antioxidant activity was evaluated in protein hydrolyzate from quinoa, using Supercritical CO_2_ extraction and ethanol as cosolvent. Antioxidant activity of protein hydrolyzate from quinoa (*Chenopodium quinoa* Willd.)

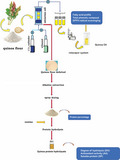

## 1 INTRODUCTION

Quinoa (*Chenopodium quinoa* Willd.) is a grain of the Chenopodiaceae family; it was a very important crop for the Incas who called it “the mother grain.” Currently, it grows mainly in the Andean region from Colombia to the northern Argentina, with Peru and Bolivia being the most important producers (Repo‐Carrasco, 2014).

Previous studies have shown the high nutritional quality of quinoa protein. It has a protein content of around 10%–22% depending on its genotype, and a greater biological importance than casein and soy protein, both proteins considered good sources of amino acids (Abugoch et al., 2008). It is also high in lysine compared to other cereals such rice and wheat has a high level of amino acids (Guerrero et al., 2015). Quinoa seeds are a good source of dietary fiber and unsaturated fats. Furthermore, it contains vitamins and minerals and is a rich source of phenolic compounds, especially flavonoids (Carciochi et al., 2014). Food and Agriculture Organization of the United Nations (FAO) called the quinoa as the food of the future due to its contribution to world food safety in the 21st century (Guerrero et al., 2015).

The demand of quinoa has increased in recent years. In Peru, its export value has increased during the last 5 years from US$ 82.206.357 in 2013 to US$ 125 430 720 in 2018 (PROMPERU, 2018); however, approximately 80% of the exported volume is in the form of grain without added value. The lifestyle of consumers around the world has changed. Large and complex economic, social, cultural, and political movements have led to a trend to change the consumption habits (Brasil Food Trends, 2020). The search for healthier life practices has led the industrial sector to develop products that promote health with comfort and high quality, in addition to adopting sustainable and clean processes (Amaral et al., 2017). Currently, it is recognized the importance of the role of proteins as physiologically active components because it is a source of bioactive peptides (Korhonen & Pihlanto, 2006). However, peptides are inactive in the original protein, requiring a process of intestinal hydrolysis or digestion to switch to their active forms (Chakrabarti et al., 2018). Enzymatic hydrolysis of the dietary protein is performed to release peptides that could have pharmacological properties such as antioxidant capacity (Udenigwe & Aluko, 2012) and physiological effects on the cardiovascular system (antihypertensive, antioxidant, antithrombotic, and hypocholesterolemic) contributing to prevention of coronary diseases (Hernandez‐Ledesma et al., 2011).

The digestibility and lipid peroxidation activity of the quinoa protein concentrate were determined in a model of zebrafish larvae, concluding that the digest obtained at the end of the digestive process showed a percentage inhibition of 82.10%, comparable to that shown by using BHT as a positive control (87.13%) (Vilcacundo et al., 2017), the antioxidant activity was determined in white, red, and black quinoa, germinated and not germinated, determining that germinated quinoa can be used in new products for its antioxidant potential and protein quality (Piñuel et al., 2019). Additionally, seventeen bioactive peptides derived from quinoa proteins have been identified, and these proteins can be used as new ingredients in the development of functional or nutraceutical food, with the aim of reducing diseases associated with oxidative stress, including cancer (Vilcacundo et al., 2018). Therefore, there is an increasing trend to use bioactive peptides, those derived from dietary proteins as intervention agents against chronic diseases.

Antioxidant peptides can also limit the oxidative damage both in prepared food (used as natural antioxidants) or by protecting the body's cells from oxidation when it is consumed in the diet (Vioque & Millan, 2005). Conventional and nonconventional vegetables as rich sources of proteins provide bioactive peptides with antioxidant capacity. Conventional sources include soybean (*Glycine max* L.), rice (*Oryza sativa* L.), corn (*Zea mays* L.), and chickpea (*Cicer arietinum* L.), while nonconventional sources include amaranth (*Amaranthus spp*.), buckwheat (*Fagopyrum esculentum* Moench), rapeseed (*Brassica napus* L.), and Mexican “Piñón” (*Jatropha curcas* L.) (Gallegos et al., 2013). A previous study has reported the extraction of quinoa peptides by enzymatic hydrolysis, for the evaluation of antioxidant capacity (Cisneros, 2017), their biological effects indicate that they are ideal for their use in the human diet (Meneguetti et al., 2011).

However, foods are complex structures that can contain different bioactive compounds, such as phenolics that have different biological activities (antioxidant, antihypertensive, antidiabetic, and antimicrobial). These compounds could also be present in hydrolysates from quinoa; therefore, it is recommendable to extract those phenolic compounds in previous stages of enzymatic hydrolysis to avoid interferences during the evaluation of quinoa peptides. To separate phenolic compounds from proteins derived from various food matrices, extraction methods applied the use of solvents such as ethanol or acetone, clean technologies such as supercritical extraction, ultrasound‐assisted extraction and extraction with constant pressure water (Banan‐Mwine et al., 2017).

Likewise, the sample must be prepared for the extraction of quinoa protein, to reduce and standardize the grain size (milling and shifting) as well as to eliminate the oil with the application of solvents such as hexane or petroleum ether (Guerrero et al., 2015). However, supercritical fluids extraction technology (SCF) is an alternative method of recovering vegetables extracts, which generally allows obtaining high‐quality extracts compared to conventional methods (Wejnerowska & Ciaciuch, 2018). Supercritical carbon dioxide (CO_2_) is the most used solvent in food extractions because it is not toxic, not explosive, inexpensive, it is separated easily and completely from the extract, and it has the potential for selective extractions by varying pressure and temperature (Przygoda & Wejnerowska, 2014).

The fortification of products uses peptides; these are market as nutraceuticals or functional food. They are also used in the cosmetic industry to promote skin health, dermatological purposes (Agyei et al., 2016; cited by Cisneros, 2017).

Quinoa seeds are an excellent source of antioxidants, which have a high correlation with the content of total phenolic compounds (Tang & Tsao, 2017). However, lipophilic antioxidants in quinoa such as fatty acids, tocopherols, and carotenoids also contribute to its antioxidant activity. Therefore, it is important to carry out a previous separation of these hydrophilic and lipophilic compounds to obtain antioxidant capacities directly related to quinoa peptides. The objective of this study was to evaluate the influence of the type of pretreatment (conventional method with organic solvents versus extraction using supercritical fluids) on the antioxidant activity of quinoa protein hydrolysate.

## 2 MATERIALS AND METHODS

### 2.1 Reagents

The gallic acid standards, Folin–Ciocalteu reagent, 2,2‐diphenyl‐1‐picrylhydrazyl, 2,4,6‐tris (2‐pyridyl)‐*s*‐triazine (TPTZ), and Trolox were purchased from Sigma‐Aldrich. Experimental reagents such as sodium acetate, petroleum ether, sodium hydroxide, chlorohydric acid 37%, boric acid, ammonium sulfate, glacial acetic acid, sulfuric acid, ethyl alcohol, methyl alcohol, albumin serum bovine, acetic anhydride, sodium carbonate, and ferric chloride were obtained from Synth (Diadema). Deisenhofen, Germany.

### 2.2 Sample preparation

Quinoa (*Chenopodium quinoa* Willd.) seeds of Hualhuas variety were used in this study; the sample was supplied by the leguminous and cereal program of National Agrarian University La Molina, Lima, Peru. The proximate composition of quinoa (% dry basis) was moisture 10, carbohydrates 75.15, protein 13.97, fat 5.70%, and ash 3.38.

Quinoa seeds were washed with cold water during some time to remove saponins; then, they were dried at 50°C until reaching 8% of humidity. After that, they were ground in an IKA Basic 11 (USA) mill. Finally, the wheat flour was sifted in a 0.5 mm (N°35) sifter with a similar particle size.

### 2.3 Pretreatments and quinoa protein extraction

**Figure 1:**
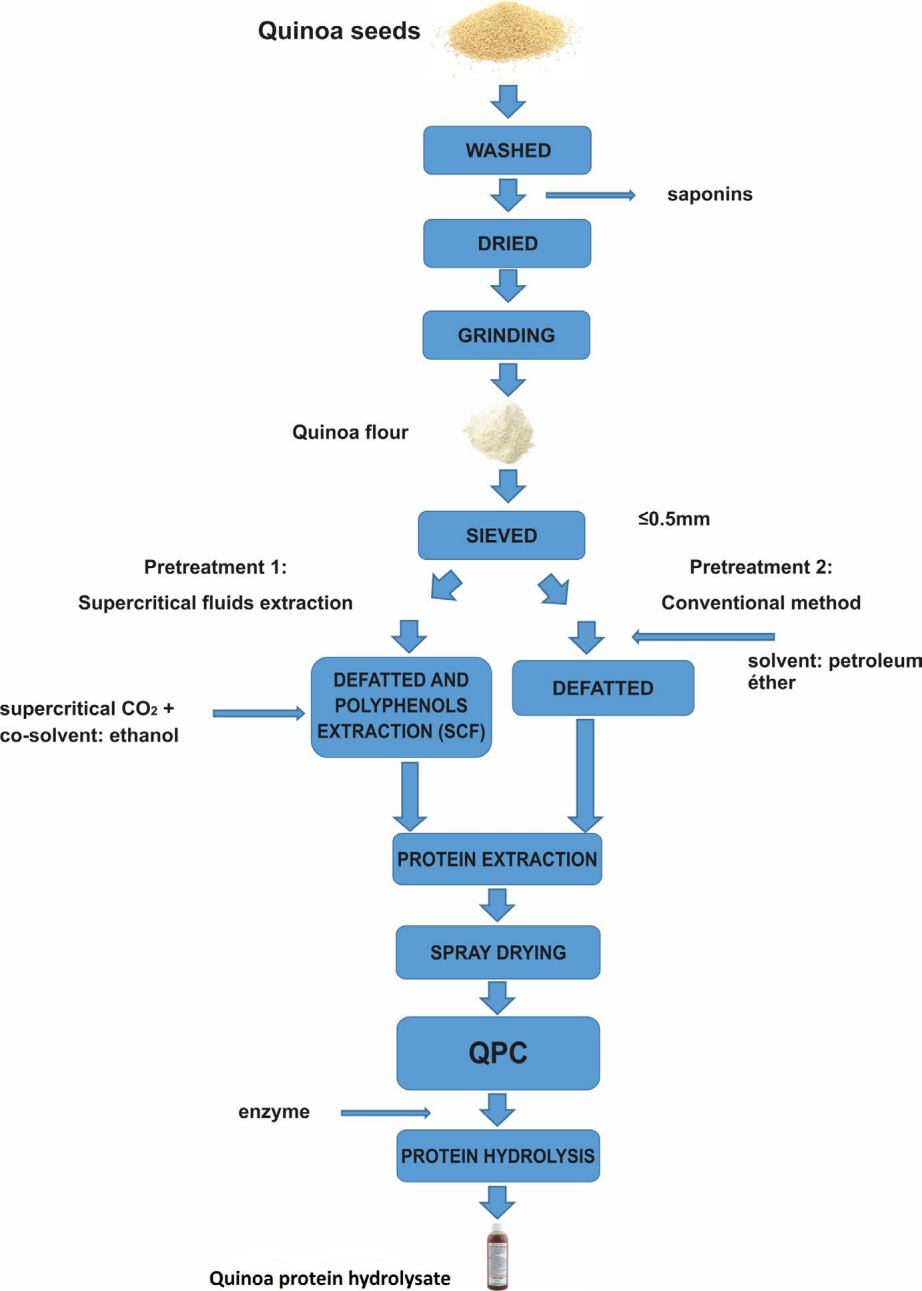
Quinoa protein hydrolysate process

For the extraction of protein from flour, the methodology proposed by Fritz et al. (2011) for proteins obtained from amaranth seeds, then applying modifications during the stage of oil removal. The flow of operations indicating the factors studied. Figure 1.

#### 2.3.1 Pretreatment of quinoa flour with supercritical fluids

The objective of pretreatment with supercritical fluids was to remove oil from quinoa flour to avoid interferences during protein extraction and extract the phenolic compounds with the aim of avoiding interferences during trials of antioxidant activity of quinoa extract hydrolysate (Banan‐ Mwine et al., 2017).

A multi‐solvent equipment (2802.0000; Top Industry) was used (it has a software used to control the temperature of the preheater, reactor, separator and to register the pressures). This equipment designed to develop different extraction techniques, among them: the extraction with supercritical CO_2_ and a polarity modifier. The extractor has a cosolvent pump that absorbs ethanol form a bottle and carries it to the reactor that contains the sample; the extraction of oil and phenolic compounds from quinoa flour compounds applied this technique. Out placing quinoa flour in the reactor cell carried the extraction; the cosolvent pump absorbed 96% ethanol, considering a sample/solvent ratio of 7 g/100 ml. The operating parameters of the equipment were *p* = 230 bar, T_reactor_ = 55°C, and Flow = 35 g CO_2_/min; the time was approximately 4 hr.

#### 2.3.2 Oil extraction using petroleum ether (conventional method)

Oil was removed from flour after shifting in a ratio of 150 g of flour to 500 ml of petroleum ether, stirring for 16 hr at refrigeration temperature. After that, the residual solvent in the product was filtered and eliminated in the dry cabin with airflow recirculation at 50°C per 1 hr.

#### 2.3.3 Extraction of quinoa protein

The same method of protein extraction was applied to each sample of quinoa flour, as described below.

Alkaline extraction: Protein extraction was carried out under a raw material‐solvent ratio of 1:10, pH 10 (using NaOH 1 N), 50°C during 30 min in bain‐marie with agitation.

Centrifugation: After alkaline extraction, the solution was centrifuged at 8150 *g* for 45 min at a temperature of 10°C, conserving the supernatants. A second extraction with the remaining sample used for reference was performed under similar conditions.

Filtering: The supernatants of both extractions were filtered with a Kitasato flask, vacuum pump using Whatman N°1 filter paper, and then, precipitation was carried out.

Isoelectric precipitation: pH was adjusted to 4.8 (using HCl 1 N), stirring during 5 min at room temperature. The precipitate obtained was conserved and separated from the supernatant.

Washed and centrifuged: The precipitates in both samples were two washes with distilled water were carried out in a concentrate/solvent ratio of 1:5, followed by centrifugation at 8150 *g* for 45 min at a temperature of 5°C, recovering the precipitation. Then, neutralization was performed in a suspension of distilled water, and the pH was regulated to 7 (using NaOH 1 N). Then, samples were spray‐dried in a laboratory‐scale spray‐dryer (Buchi B‐290), at air inlet temperature of 120°C, flow rate 600, and product feed of 15%. Finally, the protein quinoa was stored in polyethylene bags at 20°C for further determination of the total protein and hydrolysis.

### 2.4 Protein hydrolysis

**Figure 2:**
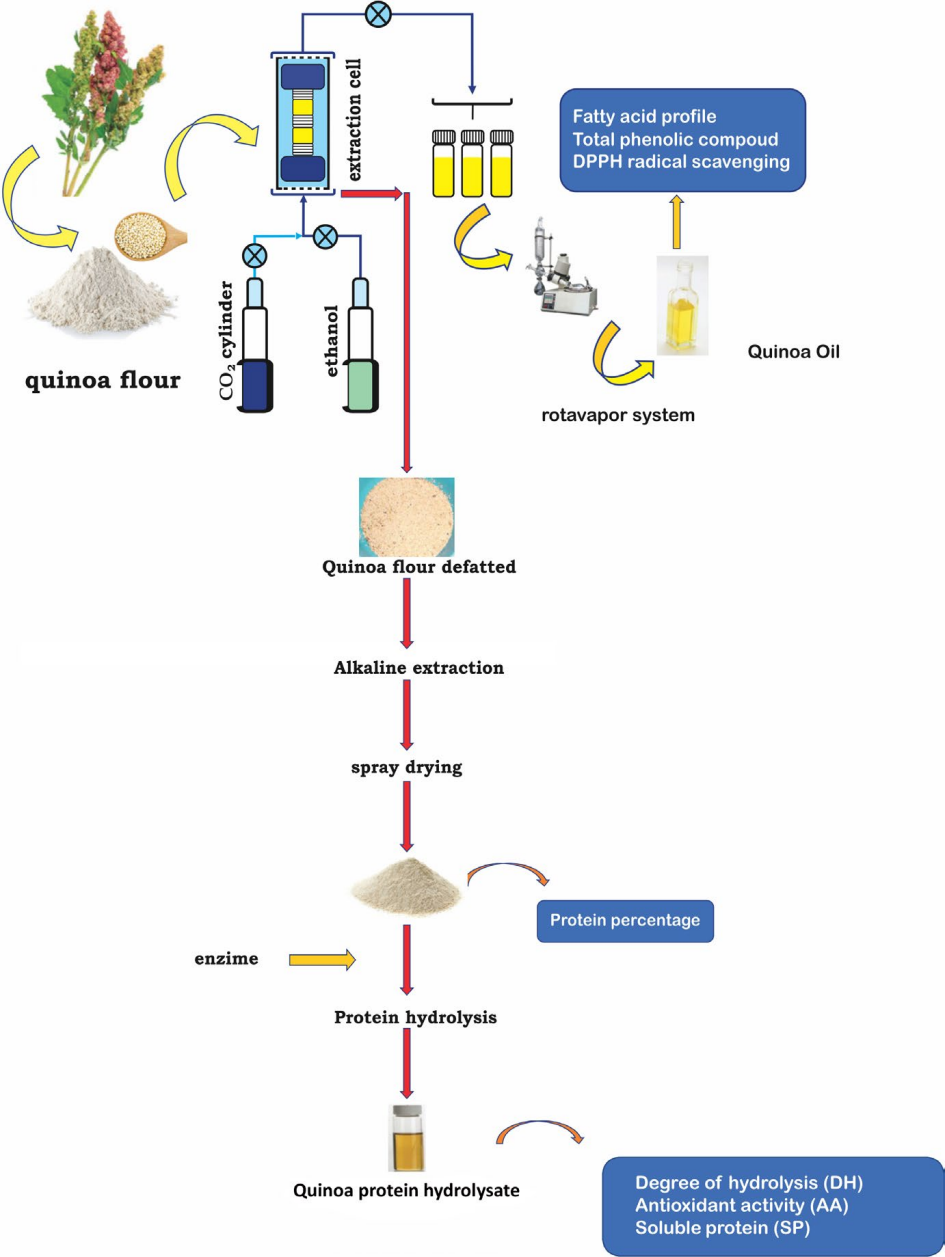
Graphical essays

Hydrolysis of quinoa protein concentrates was conducted in accordance with the proposal of Guerrero ‐ Ochoa et al. (2015); we used a commercial endopeptidase COROLASE^®^ 7089 (AB Enzymes). Obtained from *Bacillus subtilis* cultures that hydrolyses high molecular proteins into low molecular peptides. A 2.5% suspension prepared (protein was dissolved in phosphate buffer 0.2 M, pH 7.0); the suspension was heated to 55°C; then, the enzyme was added. The enzyme concentration was 4.2 UHb/g protein; suspension was maintained in gentle and constant agitation; the time was 120 min. Reaction was carried out in 250 ml Erlenmeyer. Finally, the reaction stopped by thermal inactivation at 85°C for 10 min; the mixture cooled down at room temperature, followed by centrifugation at 8150 *g* for 45 min to recover the peptides contained in the supernatant; then, we analyzed the trials. Figure 2 shows the trials performed in quinoa protein hydrolysate.

### 2.5 Sample analysis

#### 2.5.1 Determination of total fat content

Total lipids extracted by Soxhlet extraction method using hexane as solvent and then determined by gravimetry.

#### 2.5.2 Determination of total phenolic content

The method reported by Repo‐Carrasco & Encina (2008) used to determine phenolic compounds of quinoa flour, but with light modifications. Then, we performed ethanol extraction and centrifugation of efficient separation. A sample of 5 g 20 ml Methanol was placed inside a Falcon tube which was blended in a high‐speed homogenizer for 1 min until reaching a uniform consistency. We kept the homogenized sample at rest for 24 hr under refrigeration (4°C) and then centrifuged for 30 min at 5812 *g*.

The content of total polyphenols was determined using the method described by Singleton & Rossi (1965), reported by Ramos‐Escudero et al. (2012) using Folin–Ciocalteu reagent for polyphenol content determination. A sample of 100 µl of the extract reacted with 750 µl of the Folin–Ciocalteu reagent 0.2 N; 5 min after reaction, 750 µl of sodium carbonate at 7.5% was added. The reaction carried out at 25°C in darkness for 30 min. The absorbance measured at 725 nm. The calibration curve carried out with the following concentrations of gallic acid: 5, 10, 40, 70, and 100 µg/ml. The results were expressed as gallic acid equivalents (GAE) in mg/100 g of quinoa seeds; flour before and after treatment with SCF in a dry weight basis (DW).

#### 2.5.3 Determination of total protein content

The Kjeldahl methodology was used to determine the total protein in the total concentration of quinoa, according to AOAC method 984.13; (AOAC, 1995). This method based on the destruction of organic matter with sulfuric acid concentrations, forming ammonium sulfate that in the excess of sodium hydroxide releases ammonia. It is receiving in sulfuric acid and formed ammonium sulfate; then, it assessed the excess of acid with sodium hydroxide in the presence of methyl red or in boric acid, forming ammonium borate and assessed by hydrochloric acid. The protein factor conversion used was of 5.85.

#### 2.5.4 Determination of soluble protein

We measured soluble proteins according to the methodology proposed by Lowry et al. (1951). This is a colorimetric method where the proteins react with Folin–Ciocalteau reagent which is reduced by the residues of tyrosine and tryptophane present in a cuproprotein complex to give another volatile blue complex which intensity is proportional to the aromatic amino acid concentrations and will change according to the kind of proteins.

This analysis carried out mixing 400 μL of the hydrolyzed sample with 2 ml of the reaction solution of Na_2_CO_3_ at 2% in 0.1 M of NaOH, CuSO_4_.H_2_O at 0.5% in distilled water and sodium tartrate at 1% in distilled water, in a ratio of 100:1:1; 200 μl. Folin–Ciocalteau reagent 1 N was added to this mixture which was then agitated and incubated at room temperature for 30 min. After that, absorbance measured at 650 nm. The results were determined with the help of a standard curve using a BSA solution as pattern in a concentration range of 0.05 – 0.3 mg/ml. The blank was prepared by replacing the hydrolyzed sample with 400 μl of distilled water.

#### 2.5.5 Determination of the degree of hydrolysis

Hydrolysis was determined by the method reported by Adler – Nissen (1979), although some modifications were made in this investigation. In a test tube, we added 0.5 ml of borate buffer 0.2 M (pH 8.2); 0.63 ml of the hydrolyzed protein sample dissolved in SDS at 1% and 0.5 ml of picrylsulfonic acid solution (TNBS, 1 mg/ml). The entire complex, protected from light, was agitated and incubated at 50°C during 60 min at bain‐marie. The reaction was stopped adding 1 ml of HCl 0.1 N, and then, it was left to stand at room temperature for 20 min. Later, we added 2 ml of distilled water, and then, it stands other 10 min before absorbance measured at 340 nm. The results were determined with the help of a standard curve using a solution of L‐leucine in SDS at 1% in a concentration range of 0.5 – 3.0 mM. The sample used for reference was prepared substituting the sample for 0.63 ml of SDS solution at 1% in water. The values of the degree of hydrolysis (DH) calculated by the following formula:

(1) $$\text{DH}\;\left(\%\right)=\frac{100\times \left(\text{AN}2‐\text{AN}1\right)}{\text{Npb}}.$$

Where,

DH: degree of hydrolysis.

AN1: Amino nitrogen content of the protein before hydrolysis. It is the sample taken before adding the enzyme, considered as time 0 (mg/g of protein).

AN2: Amino nitrogen content of the protein after hydrolysis (mg/g of protein).

Npb: Amino nitrogen content of peptide bonds in the substrate (protein concentrate), which was determined after total hydrolysis with HCl 6 M at 110°C for 24 hr followed by filtration through Whatman N° 40 filter paper and the subsequent neutralization with NaOH 6 M.

#### 2.5.6 Determination of antioxidant activity by ABTS

We used the method reported by Torruco et al. (2009) and Re et al. (1999). It was adapted for hydrolyzed proteins in this investigation. We performed this trial preparing solution A: 7 mM of ABTS and solution B: 25.4 mM of K2S_2_O_8_, both diluted in deionized water. We mixed in a ratio of 9:1 and stored for 12–16 hr in darkness before their use. This solution name is mother solution.

After that time, the diluted solution of ABTS was prepared in phosphate buffer saline (PBS), which is composed of 8.18 g/L of NaCl, 0.27 g/L of KH_2_PO_4_, 1.42 g/L of Na_2_HPO_4_, and 0.15 g/L of KCl dissolved in deionized water until reaching a pH of 7.4 (using NaOH 1 M). After that, the diluted solutions of ABTS and PBS mixed at a ratio of 1:50, respectively, until reaching an absorbance from 0.70 ± 0.03 to 734 nm. For measuring antioxidant activity, 40 μL of the hydrolyzed sample previously diluted in PBS was used, which was then mixed with 4 ml of the diluted solution of ABTS. An hour after the reaction (in darkness), absorbance was determined at 734 nm. The results were determined through a standard curve using a Trolox solution in a concentration range of 0.5–2.0 mM and expressed in μmol of Trolox equivalents (TE)/g protein. Similarly, the sample used as reference was prepared using PBS as replacement of the sample.

### 2.6 Statistical analyses

All the assays were conducted in triplicate. The results were expressed as mean ± standard deviation and analyzed using SPSS for Windows version 24.0 (SPSS, Inc., Chicago, IL, USA). The means compared by one‐way ANOVA followed by Tukey's post hoc test (*p* < .05).

## 3 RESULTS AND DISCUSSION

### 3.1 Comparing the effects of pretreatment by conventional Petroleum ether extraction (Soxhlet method) and SCF on the previous stages of enzymatic hydrolysis

#### 3.1.1 Effect of extraction with petroleum ether (Soxhlet method) and SCF in the percentage on total fat of quinoa flour

The total fat content of the quinoa flour sample (QF) for both pretreatments was 5.70%, the pretreatments applied, determining that the amount of fat in the flour pretreated with petroleum ether (DQE) was 1.58%, and for quinoa pretreated with SCF, it was 0.03%; in other words, the quantity of fat removed is 77.59% (DQE) and 99.7% (DQF), respectively. Therefore, the type of pretreatment has a significant effect on the defatting process (*p* < .05). Wejnerowska and Ciaciuch (2018) report a percentage of recovered oil of 89% using SCF and 20% methanol/ethanol as cosolvent (1:1 p/p) and a particle size of 0.5 mm. Additionally, in the same study, the percentage of recovery is higher than the ones obtained by conventional methods (Soxhlet). In general, a greater recovery evidenced and compared to the conventional pretreatment.

On the other hand, the fatty acid profiles and bioactive compounds of oil extract recovered in the extraction with supercritical fluids by GC‐FID and UV‐vis spectroscopy analyzed. Wejnerowska et al. (2018) report higher values in fatty acids C16.0 (11.2%), C18:1 (28.2%), and C18:2 (50.9%) which may be due to the variety used because, in the same study, it is evidenced that regardless of the method and extraction conditions, they do not affect the composition of fatty acids significantly. Additionally, Tang and Tsao (2017) reported the content of fatty acids of quinoa grains is principally linoleic (52%), oleic (25%), palmitic (10%), and α‐linolenic (4%) acids. The total phenolic content assessed by Folin–Ciocalteu reagent was 34.28 mg GAE/100 g, and the amount of sample mass required to inhibit 50% of DPPH free radicals was 27%. Results—Table 1.

**Table 1:** Characteristics of quinoa oil extract obtained by supercritical CO_2_ and ethanol as cosolvent

Fatty acid (% of total fatty acids)							TPC (mg GAE/100 g)	DPPH radical scavenging (%)
C14:0	C16:0	C18:0	C18:1w‐9	C18:1w‐7	C18:2w‐6	C18:3w‐3		
0.31	10.01	0.68	23.53	0.94	49.56	3.69	34.28	27

Note

Extraction with supercritical CO_2_ mass flow of 35 g/min at 23 MPa, 55°C, for 240 min, addition of ethanol as cosolvent 7–8 g of quinoa/100 ml; quinoa flour particle size was 500 µm.

Abbreviations: TPC, total phenolic content.

#### 3.1.2 Effect of extraction with petroleum ether (Soxhlet method) and SCF on the Total protein of quinoa protein concentrate

The total protein content in the quinoa flour (QF) was 13.97%. After applying the two pretreatments, the alkaline extraction of the protein from both samples was carried out, the quinoa protein concentrate (QPC) was obtained by Spray drying method, the total protein content in the quinoa protein concentrate pretreatment with petroleum ether (QPC‐E) being 72.03%, and the quinoa protein pretreatment with supercritical fluids (QPC‐F) was 72.18%.

The type of pretreatment does not have a significant effect on the protein content of QPC‐E and QPC‐F, respectively (*p* < .05), using a conversion factor of 5.85, being within the range reported by Cisneros, 2017 who obtained a value of 73.24% using quinoa Huallhuas variety and pretreatment with petroleum ether. The value of the total protein for each type of pretreatment is within the range reported by Abugoch James (2009), which is 72.2%–83.5% and surpasses the one mentioned by Aluko and Monu (2003) (65.5%). Guerrero et al. (2015) optimized the process of extraction of quinoa protein using hexane as. solvent for fat removal obtaining 62.5% of protein, value that was lower than the one obtained in this study.

#### 3.1.3 Effect of extraction with petroleum ether (Soxhlet method) and SCF on total phenolic compound of quinoa protein concentrate

The initial content of phenolic compounds in the quinoa flour (QF) was 117.97 mg GAE/100 g; then, both pretreatments were applied, determining that the content of phenolic compounds in the quinoa flour pretreated with supercritical fluids was (QF‐F) was 51.67 mg GAE/100 g. Then, the protein was extracted by alkaline hydrolysis for both pretreatments, then the phenolic compounds content was determined for QPC‐E was 41.17 mg GAE/100 g, and for QPC‐F was 15.17 mg GAE/100 g.

The content of phenolic compounds of the extracted oil was 34.28 mg GAE/100 g. In this context, the type of pretreatment has a significant effect on the residual total phenolic compounds levels in QF and QPC (*p* < .05). To date, no study that uses pretreatment with supercritical fluids reported; nevertheless, other investigations were conducted using supercritical CO_2_ and methanol, ethanol, and methanol/ethanol as cosolvents, demonstrating that the addition of polar cosolvents improves the efficiency of the extraction. However, Carciochi et al, 2014. report a higher value of recovery of 102.86 mg GAE/100 g, using an extraction at a temperature of 60°C, 80% ethanol as solvent and without application of ultrasound. On the other hand, Nickel et al. (2016) carried out a study about the effects of different types of processing on total component of phenolic compounds and antioxidant capacity, demonstrating that other processes such as washings with running water and cooking with boiling water increase the content of phenolic compounds and antioxidant capacity.

It has been observed that the application of the supercritical fluids such as CO_2_ and ethanol as cosolvent has a significant effect (*p* < .05) on the reduction of the quantity of fat and total phenolic compounds, achieving a higher degree of purification of the sample before protein extraction and enzymatic hydrolysis (data not shown).

### 3.2 Comparing the effects of pretreatment by conventional petroleum ether extraction (Soxhlet method) and SCF on antioxidant activity of quinoa protein hydrolyzed

The type of pretreatment does not have a significant effect on the soluble protein of QPC‐E and QPC‐F, respectively (*p* < .05); however, the enzymatic hydrolysis increases the soluble protein of the hydrolyzed quinoa protein hydrolyzed (QPH)‐E (7.18 mg/ml) and QPH‐*F* (7.02 mg/ml); these values showed significant differences (*p* < .05). The effects revealed that protein hydrolysis dissociates insoluble protein aggregates, produces smaller peptides, increases exposure of hydrophilic groups, and facilitates interaction of hydrophilic amino acids with the aqueous environment (Aluko & Monu, 2003).

The degree hydrolysis is an important parameter for the determination of functional and bioactive properties of protein hydrolysates, Sila and Bougatef (2016). The conditions of hydrolysis such as time, temperature, pH, and enzyme activity have a great influence on this parameter (Cisneros, 2017).

Agyei et al. (2016) mention that a DH greater than 10 percent allows obtaining bioactive peptides for medical and food applications. Enzymatic hydrolysis of both samples performed, Table 2 also presented for comparison between study groups, showing a significant difference on the degree of hydrolysis, which is slightly higher than the one obtained in the sample with conventional pretreatment (*p* < .05). These values are closer to the ones reported by Mahdavi et al. (2018) that corresponded to 24.65%. However, Guerrero et al. (2015) reported an approximate value of 30% during the same period as the one used in this study (120 min). Other studies of quinoa protein hydrolyzed, report a higher degree of hydrolysis (36%) using pepsin for the hydrolysis at 120 min (Shi et al., 2019).

**Table 2:** Antioxidant activity (AA), degree of hydrolysis (DH), and soluble protein (SP) in quinoa protein hydrolyzed pretreated with petroleum ether and SCF

	QPE	QPH‐E	QPF	QPH‐F	*p*‐Value
SP (mg/ml)	6.82 ± 0.04^c^	7.18 ± 0.04^a^	6.89 ± 0.02^c^	7.02 ± 0.05^b^	<.01
DH (%)	0.000^c^	24.33 ± 0.04^a^	0.000^c^	23.93 ± 0.04^b^	<.01
AA (µmol TE/g protein)	1,046.02 ± 10.92^c^	1,448.84 ± 8. 40^a^	875.51 ± 12.13^d^	1,181.64 ± 13.25^b^	<.01

Note

* One‐way ANOVA (*p*<.05). Values (mean ± *SD*, *n* = 3) in the same row with different letters (a–c) are significantly different (*p* < .05) (“Tukey's Multiple Comparison Test”).

Abbreviations: QPE, Quinoa protein pretreated with petroleum ether; QPF, Quinoa protein pretreated with supercritical fluids; QPH‐E, Quinoa protein hydrolysate pretreated with petroleum ether; QPH‐F, Quinoa protein hydrolysate pretreated with supercritical fluids.

In this context, it demonstrates that the enzyme used, and the hydrolysis conditions have been appropriate. Consequently, the differences are due to the type of enzyme used, Segura et al. (2013), cited by Cian et al. (2015), mention that when endoproteases are used, DH values of no more than 20–25 percent are reached.

The type of pretreatment showed a significant effect on the antioxidant activity (AA) QPH‐E and QPH‐F, respectively (*p* < .05), also increases with enzymatic hydrolysis in both pretreatments. The highest AA increased (38.50%) was for conventional method, compared to (34.96%) for pretreatment with supercritical fluids. Similar studies report close values of Antioxidant activity at 180 min (775 mmol Trolox equivalents/mg protein), from quinoa protein hydrolyzate, using *Bacillus* spp. Alkaline serine protease (Peña et al., 2017).

As it is stated by Tang and Tsao (2017), this higher antioxidant can be due to a remaining of total phenolic compounds and lipophilic antioxidants such as fatty acids, tocopherols, and carotenoids that also contribute to the antioxidant activity and could be extracted by conventional pretreatment (Banan‐ Mwine et al., 2017).

Flavonoids and phenolic acids from quinoa seeds performed by HPLC, and the main phenolic acid found was gallic acid. p‐Hydroxybenzoic acid, vanillic acid, p‐coumaric acid, caffeic acid, and cinnamic, but the main flavonoid found in the seeds was orientin and rutin (Pasko et al., 2008). In the present study, the content of flavonols was analyzed by spectrometry on the quinoa protein hydrolyzates, finding a higher amount of flavonols expressed in mg rutin equivalent/ 100 g on the sample with conventional pretreatment than the sample with supercritical fluids pretreatment (results not showed).

## 4 CONCLUSION

The results obtained showed a significant effect the type of pretreatment on the antioxidant activity of QPH, the AA in QPH‐E was higher than QPH‐F, and this is due to the presence of phenolic compounds. The application of SCF to separate phenolic compounds in previous stages of enzymatic hydrolysis, it is a good alternative to avoid interference when determining biological activities.

Finally, supercritical CO_2_ is a solvent with unique properties with regard to other solvents; it is nonflammable, nontoxic, economic, not expensive, nonpolar, and easy to mix; obtaining ingredients free of contaminants and conserving its nutritional and functional properties as well as a maximum use of the extractable and nonextractable material. This aligned with the new tendencies of the food industry such as the production of natural and functional ingredients, sustainable foods, foods of high nutritional value, and foods with clear labels.
